# Natural Product Skatole Ameliorates Lipotoxicity-Induced Multiple Hepatic Damage under Hyperlipidemic Conditions in Hepatocytes

**DOI:** 10.3390/nu15061490

**Published:** 2023-03-20

**Authors:** Sin-Hyoung Hong, Yeonhee Hong, Minji Lee, Byeong-Rak Keum, Gun-Hwa Kim

**Affiliations:** 1 Division of Research Center for Bioconvergence Analysis, Korea Basic Science Institute, Cheongju 28119, Republic of Korea; hongsi8493@gmail.com (S.-H.H.); hyhbona@naver.com (Y.H.); mmcc0101@naver.com (M.L.); br0104@postech.ac.kr (B.-R.K.); 2 Department of Bio-Analytical Science, University of Science and Technology, Daejeon 34113, Republic of Korea; 3 Research Center for Drug Development, CYPHARMA, Daejeon 28119, Republic of Korea; 4 Department of Life Sciences, Pohang University of Science and Technology, Pohang 37673, Republic of Korea; 5 Department of Analytical Science and Technology, Graduate School of Analytical Science and Technology (GRAST), Chungnam National University, Daejeon 34134, Republic of Korea

**Keywords:** skatole, natural products, nonalcoholic fatty liver disease, lipotoxicity, lipoapoptosis, hepatic damage, lipid accumulation, endoplasmic reticulum stress, oxidative stress

## Abstract

Skatole (3-methylindole, 3MI) is a natural-origin compound derived from plants, insects, and microbial metabolites in human intestines. Skatole has an anti-lipid peroxidation effect and is a biomarker for several diseases. However, its effect on hepatocyte lipid metabolism and lipotoxicity has not been elucidated. Hepatic lipotoxicity is induced by excess saturated free fatty acids in hyperlipidemia, which directly damages the hepatocytes. Lipotoxicity is involved in several metabolic diseases and hepatocytes, particularly affecting nonalcoholic fatty liver disease (NAFLD) progression. NAFLD is caused by the accumulation of fat by excessive free fatty acids (FFAs) in the blood and is accompanied by hepatic damage, such as endoplasmic reticulum (ER) stress, abnormal glucose and insulin metabolism, oxidative stress, and lipoapoptosis with lipid accumulation. Hepatic lipotoxicity causes multiple hepatic damages in NAFLD and has a directly effect on the progression from NAFLD to nonalcoholic steatohepatitis (NASH). This study confirmed that the natural compound skatole improves various damages to hepatocytes caused by lipotoxicity in hyperlipidemic conditions. To induce lipotoxicity, we exposed HepG2, SNU-449, and Huh7 cells to palmitic acid, a saturated fatty acid, and confirmed the protective effect of skatole. Skatole inhibited fat accumulation in the hepatocytes, reduced ER and oxidative stress, and recovered insulin resistance and glucose uptake. Importantly, skatole reduced lipoapoptosis by regulating caspase activity. In conclusion, skatole ameliorated multiple types of hepatocyte damage induced by lipotoxicity in the presence of excess free fatty acids.

## 1. Introduction

Nonalcoholic fatty liver disease (NAFLD) is caused by increased levels of free fatty acids (FFAs) due to metabolic diseases such as obesity and diabetes. It is a progressive liver disease caused by de novo lipogenesis [1]. NAFLD is the most common liver disease worldwide and is estimated to affect more than 30% of the population [2]. Patients with NAFLD have increased levels of serum FFAs [3]. Moreover, FFAs induce excessive lipid accumulation in the liver, which is closely related to the pathogenesis of NAFLD [4].

Hepatic lipotoxicity occurs in cells with excess amounts of toxic lipids; therefore, circulating FFAs directly promote hepatic lipotoxicity [3]. In response to excess FFAs, hepatocytes undergo hepatic de novo lipogenesis. Subsequently, FFAs activate oxidative mechanisms such as lipid peroxidation [5] and induce oxidative stress [6,7] by producing reactive oxygen species (ROS) [8]. The hepatic lipotoxicity mechanism involves endo-plasmic reticulum (ER) stress and lipoapoptosis. FFAs directly induce protein unfolding and stimulate biosensors involved in ER stress such as PKR-like endoplasmic reticulum kinase (PERK) in the endoplasmic reticulum to induce nucleus signaling 1 (IRE1α), activating transcription factor 6 (ATF-6) [9], and the expression of these proteins is regulated by the binding immunoglobulin protein (BiP) [10]. When FFAs induce ER stress, c-Jun N-terminal kinases (JNK) increase C/EBP homologous protein (CHOP) levels in hepatocytes [11,12]. Continuous intracellular ER stress activates apoptotic pathways such as caspase cleavage, and which leads to lipoapoptosis in hepatocytes [13,14]. Tumor necrosis factor-alpha (TNF-α) is stimulated by FFAs, the decrease in anti-apoptotic protein B-cell lymphoma 2 (Bcl-2), and the increase in pro-apoptotic protein Bcl-2-associated X protein (Bax) and upregulation of caspase activity results in apoptosis [15]. Poly-ADP-ribose-polymerase (PARP) is related to lipoapoptosis mechanisms [16,17], and interleukin 6 (IL-6) and p38 mitogen-activated protein kinases (p38) initiate an inflammatory signaling pathway in response to FFAs [18]. Furthermore, increased FFA levels in the blood induce insulin resistance (IR) and gluconeogenesis. This leads to reduced glucose uptake in the liver [19] and induction of de novo lipogenesis in hepatocytes and steatosis characteristics resulting from excessive lipid accumulation [20]. FFA-induced hepatic lipotoxicity and subsequent ER stress, lipoapoptosis signaling, and inflammatory responses promote the progression of NAFLD to nonalcoholic steatohepatitis (NASH) [21]. The current therapeutic strategies targeting NAFLD aim to treat endoplasmic reticulum stress, oxidative stress, inflammation, and apoptosis induced by lipotoxicity, but there are no available therapeutic agents for the fundamental causes of lipotoxicity [22].

Palmitic acid (PA) is a representative composition of saturated FFAs in the human body [23] and is frequently used in the research of steatosis in the liver [24]. Since PA induces abnormal accumulation of lipids in hepatocytes and the development of hepatic steatosis in NAFLD [25], several previous studies have used PA-treated hepatocytes to induce various kinds of damage seen in NAFLD, including lipotoxicity [26,27,28].

Skatole is a naturally produced biologically active compound derived from the leaves of plants including jasmine [29] and Tecoma stans [30]. In addition, skatole is a microbial metabolite and is used as an ingredient in perfumes [31]. In humans, skatole is produced during tryptophan metabolism by bacteria in the intestines of vertebrates and is a component of feces and saliva in the intestines [32,33]. According to several studies, skatole levels are used as a biomarker to detect various pathological conditions in the human body [34], such as colorectal cancer [35], irritable bowel syndrome [36], and schizophrenia [37] by detecting the concentration of skatole and its metabolites in human urine or fecal samples. Some studies have reported that exposure to skatole causes pneumotoxicity [38,39] in bronchiolar exocrine cells. Although these studies have shown that toxicity occurs with high skatole concentrations, skatole has an antioxidant effect that prevents lipid peroxidation in the pulmonary alveolar epithelium [40]. Studies on skatole-induced toxicity in the lungs have been limited to rodents and ruminants in vivo [41,42], and there is a lack of research on the toxic effects of skatole on hepatocytes and its therapeutic potential.

In the present study, we investigated the potential protective effect of skatole on hepatocytes in hyperlipidemic conditions and evaluated whether skatole modulates functions related to hepatic lipotoxicity-induced types of damage in FFA-stimulated hepatocytes.

## 2. Materials and Methods

### 2.1. Cell Culture, Preparation of Compounds, and Treatment

HepG2 cells were purchased from ATCC (Manassas, VA, USA), and SNU-449 and Huh7 cells were purchased from the Korean Cell Line Bank (Seoul, Korea). All cells were cultured in Dulbecco’s modified Eagle’s medium (Hyclone, Logan, UT, USA) supplemented with 10% fetal bovine serum (Gibco, Grand Island, NY, USA) and 1% penicillin/streptomycin (Hyclone) at 37 °C in a humidified environment containing 5% CO_2_. Skatole (purity ≥ 98%) was purchased from Selleckchem (Houston, TX, USA), and PA was prepared by conjugation of sodium palmitate (Sigma-Aldrich, St Louis, MO, USA) and fatty-acid-free bovine serum albumin (Sigma-Aldrich) according to Sigma-Aldrich’s instructions. A concentration of 0.25 mM PA was used to induce lipotoxicity-related damage by previously described methods [26,43].

### 2.2. Cell Viability Assay

Cells were seeded at a density of 2 × 10^4^ cells/well in a 96-well plate and incubated at 37 °C in CO_2_ for 18 h before treatment. To determine the cytotoxicity effect of skatole, cells were treated with different concentrations of skatole (0, 1, 5, 10 μΜ) for different time periods (6, 12, 24, 48 h). To determine the cytotoxicity effect of FFAs and skatole, hepatocytes were treated with 0.25 mM PA and 1–10 μΜ skatole were treated for 24 h. All cells were incubated at 37 °C in atmosphere of CO_2_. After treatment, the Cell Counting Kit-8 (CCK-8) (Dojindo Molecular Technologies, Rockville, MD, USA) was added to the PA and skatole treated hepatocytes and additionally incubated for 1 h. Finally, the optical density was evaluated using SpectraMax M4 plate reader (Molecular Devices, San Jose, CA, USA) at 450 nm. Cell viability was normalized to the value of the control group and shown as a percentage.

### 2.3. Endoplasmic Reticulum Staining

Cells were seeded at a density of 2 × 10^4^ cells/well in a 96-well plate and incubated for 18 h before treatment. After treatment with PA and skatole, cells were stained with ER-Tracker for 1 h at 37 °C in an atmosphere of 5% CO_2_. The nuclei were stained with 0.1 µg/mL Hoechst 33342 (ChemoMetec, Bohemia, NY, USA) for 5 min at 25 °C and examined using an Opera Quadruple Enhanced High Sensitivity (QEHS) microscope (PerkinElmer, Boston, MA, USA) with an objective at 20× magnification, and the relative fluorescence was quantified using Columbus software (PerkinElmer, Boston, MA, USA).

### 2.4. Reactive Oxygen Species (ROS) Production

To measure ROS production, hepatocytes were seeded at a density of 2 × 10^4^ cells/well in a 96-well plate and incubated for 18 h before treatment. PA and skatole-treated cells and untreated cells were stained with 5 μM cell-permeant 2′,7′-dichlorodihydrofuorescein diacetate, H2DCFDA (Thermo Fisher Scientific, Cleveland, OH, USA), and 0.1 µg/mL Hoechst 33342 (ChemoMetec). Figures were obtained using the Opera QEHS microscope (PerkinElmer) with an objective at 20× magnification, and the relative fluorescence was quantified using Columbus software (PerkinElmer). 

### 2.5. Western Blotting

Cells were seeded at a density of 2 × 10^6^ cells/well in a 6-well plate and harvested 24 h after treatment with 0.25 mM PA and 5 μΜ skatole. Total proteins were isolated from the PA and skatole treated hepatocytes using RIPA lysis extraction buffer (Thermo Fisher Scientific) or Phospho-safe buffer (Thermo Fisher Scientific) containing a proteinase inhibitor cocktail (Thermo Fisher Scientific) and phosphatase inhibitor cocktail (GenDEPOT, Barker, TX, USA), according to the manufacturer’s instructions. Protein concentrations in the cell lysates were determined using a bicinchoninic acid assay (BCA) protein assay kit (Thermo Fisher Scientific). Proteins (20 μg) were loaded and separated by sodium dodecyl sulfate-polyacrylamide gel electrophoresis (SDS-PAGE) gels and transferred to a polyvinylidene fluoride (PVDF) membrane (Merck, Darmstadt, Germany). After blocking with blocking solution (5% BSA/TBST) at 25 °C for 1 h, we incubated the membrane with the primary antibody at 4 °C for 18 h. The primary antibodies used are listed in Supplementary Table S1. Subsequently, horseradish peroxidase (HRP)-conjugated secondary antibodies were used to probe the membrane for 1 h at 25 °C. The proteins were visualized using ImageQuant LAS-4000 mini (GE Healthcare, Chicago, IL, USA), and the relative protein expression levels were measured using ImageJ software (US National Institutes of Health, Bethesda, MD, USA) [44].

### 2.6. Apoptosis Assay

Cells were harvested 24 h after treatment with or without 0.25 mM PA and 5 μΜ skatole and cultured in a 5% CO_2_ incubator at 37 °C. After washing in phosphate buffered saline (PBS), the pellet was re-suspended in 1× annexin V binding buffer (BD Biosciences, San Jose, CA, USA), and the cell suspension (1 × 10^5^ cells) was transferred to a new tube. The cell suspension was incubated with annexin V-FITC (BD Biosciences) and propidium iodide (PI) (Chemometec) for 15 min at 37 °C in the dark. Apoptotic cells were assessed by flow cytometry using a CytoFLEX cell sorter (Beckman Coulter, Brea, CA, USA) and CytExpert software (Beckman Coulter, Brea, CA, USA).

### 2.7. Caspase Activity

According to the manufacturer’s instructions, caspase activity was measured using the FAM-FLICA in vitro caspase detection kit (ImmunoChemistry Technologies, Bloomington, MN, USA). Cells were harvested 24 h after PA and skatole treatment and cultured in a 5% CO_2_ at 37 °C. After treatment, the pellet was re-suspended in apoptosis wash buffer, and the cell suspension (1 × 10^5^ cells) was transferred to a new tube. In the dark, the cell suspension was incubated with 1x FAM-FLICA caspase 3&7 for 1 h at 37 °C. Caspase activity was detected using an EnSpire multimode plate reader (PerkinElmer) at 488 nm emission/515 nm excitation.

### 2.8. Nile Red Staining

For the assay of lipid accumulation in PA-treated cells, the cells were seeded at a density of 1 × 10^4^ cells/well in a 96-well plate and incubated at 37 °C in an atmosphere of 5% CO_2_ for 24 h. After PA and skatole treatment, the cells were fixed with a 4% paraformaldehyde solution in PBS (Thermo Fisher Scientific) for 15 min at 25 °C. The fixed cells were incubated with 1 μg/mL Nile Red (Invitrogen, Waltham, MA, USA) at 37 °C for 10 min in the dark. Subsequently, the nuclei were stained with 0.1 µg/mL Hoechst 33342 (ChemoMetec) for 5 min at 25 °C. The images of the stained cells were obtained using the Opera QEHS microscope with an objective at 20× magnification, and the relative fluorescence was quantified using Columbus software. 

### 2.9. Lipid Peroxidation Assay

According to the manufacturer’s instructions, the intracellular malondialdehyde (MDA) concentration was measured using a Lipid Peroxidation Assay Kit (Sigma-Aldrich) in cell lysates. The MDA contents were detected using an EnSpire multimode plate reader (PerkinElmer) at 532 nm emission/553 nm excitation.

### 2.10. Glucose Uptake

Following the manufacturer’s protocol, glucose uptake was measured using a Glucose Uptake Cell-Based Assay Kit (Cayman, Ann Arbor, MI, USA). Cells were treated with 100 μg/mL 2-deoxy-2-[(7-nitro-2,1,3-benzoxadiazol-4-yl) amino]-D-glucose (2-NBDG) and incubated in PBS for 4 h. Glucose uptake was measured using an EnSpire multimode plate reader (PerkinElmer) at 485 nm emission and 535 nm excitation.

### 2.11. Statistical Analysis

All results are presented as the mean ± standard deviation (SD). One-way analysis of variance (ANOVA) was performed with Tukey’s Multiple Comparison post hoc test using GraphPad Prism 5 software (GraphPad, San Diego, CA, USA) for statistical analysis. The statistical significance was set to *p* < 0.05. At least three independent samples were performed for all experiments.

## 3. Results

### 3.1. Skatole Regulates Lipid and Glucose Metabolism in HepG2 Cells with PA Treatmnet

**Figure 1:**
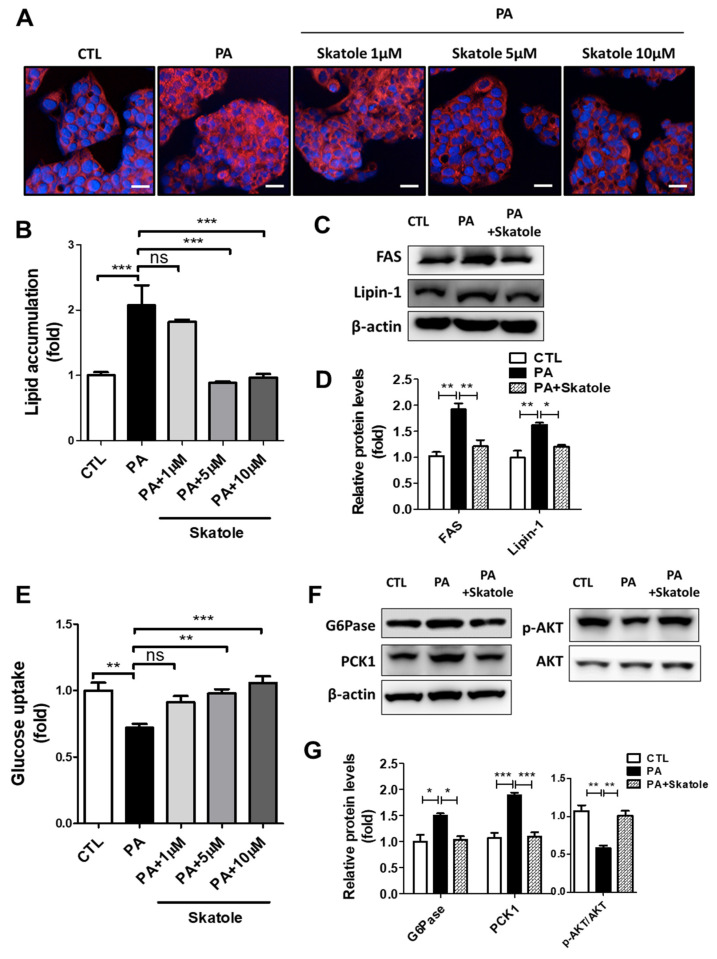
Skatole attenuates palmitic acid (PA)-induced hepatic lipid accumulation and lipogenesis-related proteins and restores glucose metabolism and insulin resistance (IR) in HepG2 cells. (**A**) HepG2 cells were treated with different concentrations of skatole (1–10 μΜ) with 0.25 mM PA for 24 h, and the lipid accumulation was analyzed using Nile Red staining. Scale bar, 25 μM. (**B**) The relative fluorescence intensity of intrahepatic lipids was analyzed using Columbus software. (**C**) HepG2 cells were treated with 5 μΜ skatole and 0.25 mM PA for 24 h. Western blot analysis indicated that skatole regulated lipogenesis-related factors. (**D**) Relative protein expression levels for lipogenesis-related factors. The relative expression levels of fatty acid synthase (FAS) and Lipin-1 were normalized with β-actin. (**E**) Glucose uptake was evaluated with 2-NBDG fluorescence intensity in HepG2 cells after the same treatment described in (**A**,**B**). (**F**) Western blot analysis indicated that skatole regulated gluconeogenesis-related factors and IR in HepG2 cells after the same treatment described in (**C**). (**G**) The relative expression levels of G6pase and PEPCK were normalized with β-actin, and p-AKT was normalized with AKT. All experiments were performed as three independent samples, and the values are shown as mean ± SD, analyzed by one-way ANOVA. (*n* = 3–5, *** *p* < 0.001, ** *p* < 0.01, * *p* < 0.05, ns: not significant. CTL: control).

In previous studies, PA-treated HepG2 cells were widely used for research on hepatic damage associated with NAFLD [27,43]. We prepared an in vitro NAFLD model in which lipotoxicity was induced by treating HepG2 cells with 0.25 mM PA for 24 h. In a preliminary study, we performed efficacy screening for hepatic steatosis using a natural product library L1400 (Selleckchem) and confirmed the association between skatole and hepatic lipid metabolism (results not shown). To clarify the effects of skatole on lipid and glucose metabolism in hepatocytes related to lipotoxicity, PA-treated HepG2 cells were exposed to different concentrations of skatole in the range of 1–10 μM for 24 h. Skatole significantly decreased lipid accumulation at a concentration of 5 μM in HepG2 cells (Figure 1A,B). Similarly, 5 μM skatole treatment had an inhibitory effect on the expression of lipogenic factors (Figure 1C,D). Subsequently, we confirmed that skatole restored the glucose uptake of HepG2 cells at concentrations above 5 μM (Figure 1E), and the expression levels of gluconeogenesis-related proteins, D-glucose-6-phosphate phosphohydrolase (G6Pase), and phosphoenolpyruvate carboxykinase 1 (PCK1), and IR-related protein phosphor-protein kinase B (AKT) were regulated by skatole treatment (Figure 1F,G). These results indicate the effect of 5 μM skatole on PA-induced abnormal lipid and glucose metabolism; thus, 5 μM skatole was used for subsequent experiments.

### 3.2. Skatole Suppressed PA-Induced ER Stress and ROS Production in HepG2 Cells

**Figure 2:**
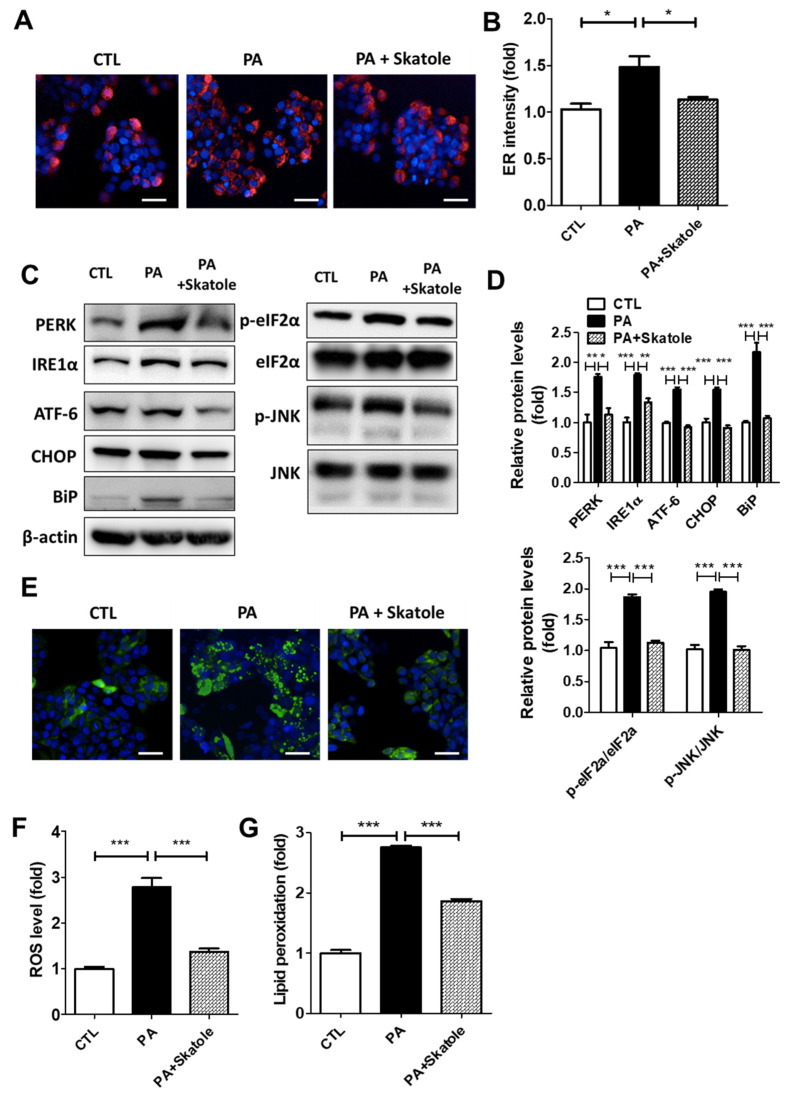
Skatole reduces PA-induced endoplasmic reticulum (ER) and oxidative stress in HepG2 cells. (**A**) ER fluorescence measured using ER-Tracker was enhanced by 0.25 mM PA and restored by treatment of 5 μM skatole in HepG2 cells exposed for 24 h. Scale bar, 25 μM. (**B**) Relative ER fluorescence intensity was measured using Columbus software. (**C**) The protein expression of PA-induced ER stress-related factors was regulated by skatole. (**D**) The relative expression levels of ER stress factors were normalized with β-actin or total protein form. (**E**) Skatole treatment attenuated PA-induced ROS production in HepG2 cells. Scale bar, 25 μM. (**F**) The fluorescence intensity of ROS was evaluated using Columbus software. (**G**) Intracellular MDA concentration of PA and skatole-treated HepG2 cells was assessed by lipid peroxidation assay kit and normalized by protein concentration. Three independent samples were performed for all experiments, and the values are shown as mean ± SD, analyzed by one-way ANOVA. (*n* = 3–5, *** *p* < 0.001, ** *p* < 0.01, * *p* < 0.05, CTL: control).

We evaluated whether skatole could control ER and oxidative stress caused by FFA-induced lipotoxicity. Initially, we investigated the effect of skatole on ER stress. Similar to a previous study that evaluated ER stress with ER fluorescence intensity in hepatocytes [45], we evaluated the regulatory effect of skatole on ER stress using fluorescence microscopy. Skatole reduced PA-induced ER intensity in HepG2 cells, suggesting that ER stress is suppressed by skatole treatment (Figure 2A,B), including reduced expression of the ER stress-related proteins, PERK, IRE1α, ATF-6, CHOP, BiP, JNK, and phosphor-eukaryotic initiation factor 2α (phosphor-eIF2α) (Figure 2C,D). Subsequently, we investigated the effect of skatole on oxidative stress. When HepG2 cells were exposed to PA, intracellular ROS levels in the HepG2 cells significantly increased compared to the control group. However, treatment with PA followed by skatole decreased ROS levels (Figure 2E,F). Next, we investigated lipid peroxidation levels by evaluating the intracellular MDA content. Similar to ROS levels, lipid peroxidation levels were downregulated by the skatole treatment of HepG2 cells (Figure 2G). These results suggest that skatole suppresses ER stress and ROS production in PA-treated HepG2 cells.

### 3.3. Skatole Attenuates PA-Induced Lipotoxicity by Reducing Apoptosis, Caspase Activity, and Inflammation in HepG2 Cells

**Figure 3:**
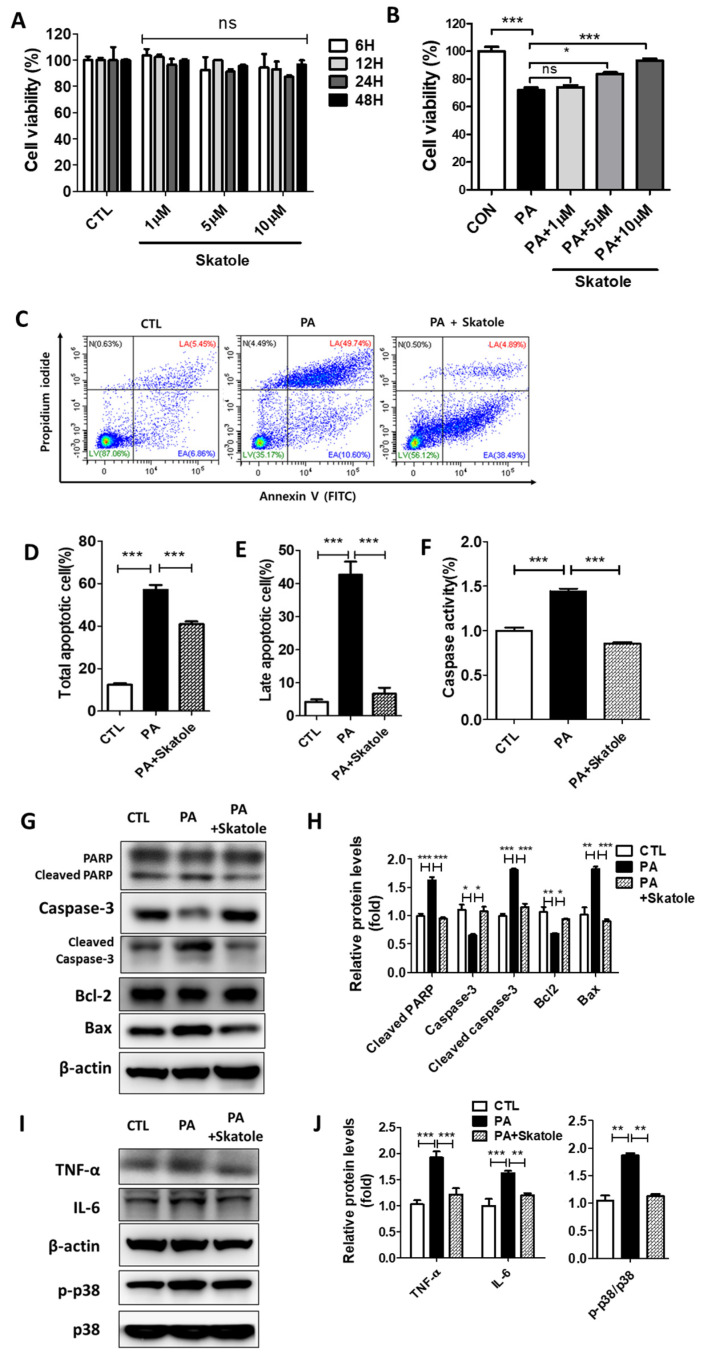
Skatole is non-toxic and inhibits PA-induced apoptosis, caspase activity, and inflammation in HepG2 cells. (**A**) HepG2 cells were treated with different concentrations (1–10 μM) of skatole for different times (6–48 h) and cell viability was analyzed. (**B**) HepG2 cells were exposed to skatole with 0.25 mM PA for 24 h and cell viability was analyzed. (**C**) HepG2 cells were treated with 0.25 mM PA and 5 μM skatole for 24 h. The apoptotic cell population was detected by flow cytometry (FCM) assay of Annexin V and PI staining detected the apoptotic cell population. (**D**) The total apoptotic cell population (Annexin V^+^) was calculated based on (**C**). (**E**) The late apoptotic cell population (Annexin V^+^/PI^+^) was calculated based on (**C**). (**F**) HepG2 cells were treated with the same conditions as (**C**). The caspase activity in HepG2 cells was measured using the fluorometric assay. (**G**) The expression levels of apoptotic proteins were estimated by Western blotting of the PA and skatole-treated HepG2 cells. (**H**) Relative apoptotic proteins were normalized with β-actin. (**I**) Inflammatory proteins were estimated by Western blot in the treated HepG2 cells. (**J**) Relative inflammatory proteins (TNF-α and IL-6) were normalized with β-actin, and phosphor-p38 was normalized with total p38. Three independent samples were performed for all experiments, and the values are shown as mean ± SD, analyzed by one-way ANOVA. (*n* = 3–5, *** *p* < 0.001, ** *p* < 0.01, * *p* < 0.05, ns: not significant. CTL: control, LV: live cells, EA: Early apoptotic cells, LA: Late apoptotic cells, N: Necrotic cells).

FFAs increase the population of apoptotic hepatocytes [46,47,48]. Thus, we evaluated changes in apoptotic parameters to clarify whether skatole attenuates lipotoxicity-induced inflammatory and lipoapoptosis signaling in hepatocytes. HepG2 cells were initially exposed to 1–10 μM skatole for up to 48 h to verify that skatole has a non-toxic effect on cells (Figure 3A). In addition, we confirmed that treatment with more than 5 μM of skatole restored the cell viability of the 0.25 mM PA-treated HepG2 cells (Figure 3B). Then, apoptotic cell populations in HepG2 cells were evaluated to determine the anti-apoptotic effect of skatole. The skatole-treated group exhibited decreased counts of total apoptotic cells and a significantly decreased number of late apoptotic cells (Figure 3C–E). In the same context, the levels of caspase activity were increased by PA treatment but were recovered to the level of the control group after skatole treatment (Figure 3F). In addition, skatole treatment decreased the expression levels of the apoptosis-related proteins, cleaved PARP and cleaved Caspase-3, and the pro-apoptotic protein, Bax (Figure 3G,H). During apoptosis, inflammatory signaling was stimulated. Therefore, we investigated the expression of inflammation-related proteins. As expected, skatole increased the expression of the anti-apoptotic protein, Bcl-2, and regulated the expression of inflammation-related proteins such as TNF-α, IL-6, and phosphor-p38 (Figure 3I,J). These results suggest that skatole treatment may significantly protect hepatocytes from PA-induced lipotoxicity, including apoptosis, caspase activation, and inflammation.

### 3.4. Skatole Is Non-Cytotoxic and Skatole Treatment Reduces ER Stress, Apoptosis, and Caspase Activity in SNU-449 and Huh7 Cells

**Figure 4:**
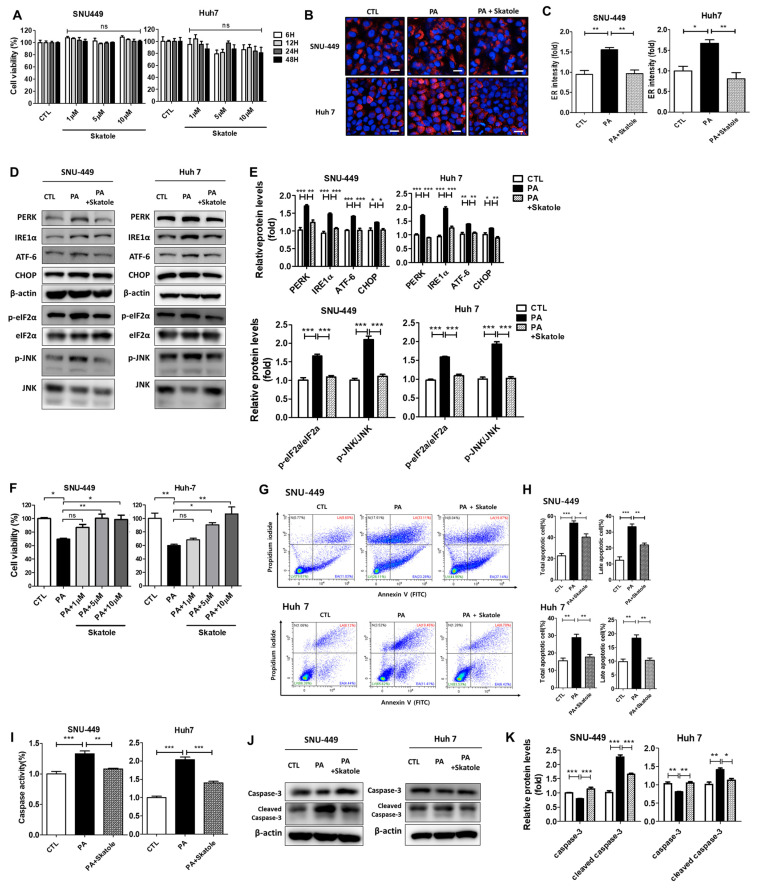
The attenuating effects of skatole on PA-induced ER stress, apoptosis, and caspase activity in SNU-449 and Huh7 cells. (**A**) SNU-449 and Huh7 cells were treated with different concentrations (1–10 μM) of skatole for different times (6–48 h), and cell viability was detected. (**B**) SNU-449 and Huh7 cells were treated with 0.25 mM PA and 5 μM skatole for 24 h. ER staining was performed using ER-tracker. (**C**) The relative ER fluorescence intensity was measured using Columbus software. (**D**) Cells were treated with the same conditions as in (**B**), and the levels of ER stress markers in treated cells were analyzed by Western blot analysis. (**E**) The relative levels of ER stress markers (PERK, IRE1α, ATF-6, and CHOP) were normalized with β-actin. Phosphor-eIF2α and phosphor-JNK were normalized to total eIF2α and JNK, respectively. (**F**) SNU-449 and Huh7 cells were treated with 0.25 mM PA and 1–10 μM skatole, and cell viability was measured. (**G**) Apoptotic cells were separated by the FCM assay of Annexin V and PI double staining. (**H**) Total apoptotic and late apoptotic cell populations were determined based on (**G**). (**I**) Cells were treated with PA and skatole with the same conditions as above. Hepatic caspase activity was evaluated using a fluorometric assay. (**J**) Apoptosis-related protein expression levels in treated SNU-449 and Huh7 cells were detected using Western blot analysis. (**K**) Relative protein levels of the apoptosis-related proteins, caspase-3, and β-actin were used for normalization. Three independent samples were performed for all experiments, and the values are shown as mean ± SD, analyzed by one-way ANOVA. (*n* = 3–5, *** *p* < 0.001, ** *p* < 0.01, * *p* < 0.05. CTL: control, LV: live cells, EA: Early apoptotic cells, LA: Late apoptotic cells, N: Necrotic cells).

PA-treated SNU-449 and Huh7 cells were used to study abnormal intracellular hepatocyte metabolism induced by excess lipid accumulation, similar to the pathogenesis of NAFLD [26,42,49,50]. To further clarify the protective effects of skatole on hepatocytes undergoing lipotoxicity, we studied its effects using two additional hepatocytes: SNU-449 and Huh7 cells. First, we confirmed that skatole was non-cytotoxic to SNU-449 and Huh7 cells. We evaluated cell viability when cells were treated with skatole alone at concentrations of 1–10 μM for up to 48 h, and skatole did not affect cell viability in either hepatocyte (Figure 4A). Then, we investigated whether skatole treatment improves ER stress, an important characteristic of hepatic lipotoxicity. The upregulated fluorescence ER intensity by PA was restored by skatole treatment (Figure 4B,C), and the protein levels of ER stress markers were also downregulated by skatole treatment in SNU-449 and Huh7 cells (Figure 4D,E). Subsequently, to determine the concentration of skatole at which cytotoxicity caused by PA exposure was ameliorated, we measured the cell viability of SNU-449 and Huh7 cells treated with 0.25 mM PA and 1–10 μM skatole for 24 h (Figure 4F). As a result, SNU-449 and Huh7 cells treated with more than 5 μM showed anti-cytotoxic effects against PA treatment. To verify the effect of skatole on apoptotic features related to lipotoxicity, the apoptotic cell population was evaluated. Similar to the HepG2 cells, the apoptotic cell population ratio induced by PA was significantly reduced by skatole in SNU-449 and Huh7 cells. In particular, the late apoptotic cell population ratio tended to considerably decrease (Figure 4G,H). Furthermore, our results confirmed that caspase activity, one of the factors involved in apoptosis, and the expressed protein levels of the apoptotic protein, caspase-3, were also reduced by skatole in SNU-449 and Huh7 cells (Figure 4I–K). Notably, in Huh7 cells, caspase activity induced by PA was more sensitive to skatole than that in HepG2 cells and was noticeably reduced by skatole treatment to the levels of the control groups. The above results indicate that skatole reduces ER stress not only in HepG2 cells but also in other hepatocytes and protects cells from lipotoxicity by regulating apoptosis-related signaling such as that of caspase-3, thereby reducing apoptosis.

### 3.5. Skatole Treatment Reduces Lipid Accumulation and ROS Production and Enhances Glucose Uptake in SNU-449 and Huh7 Cells

**Figure 5:**
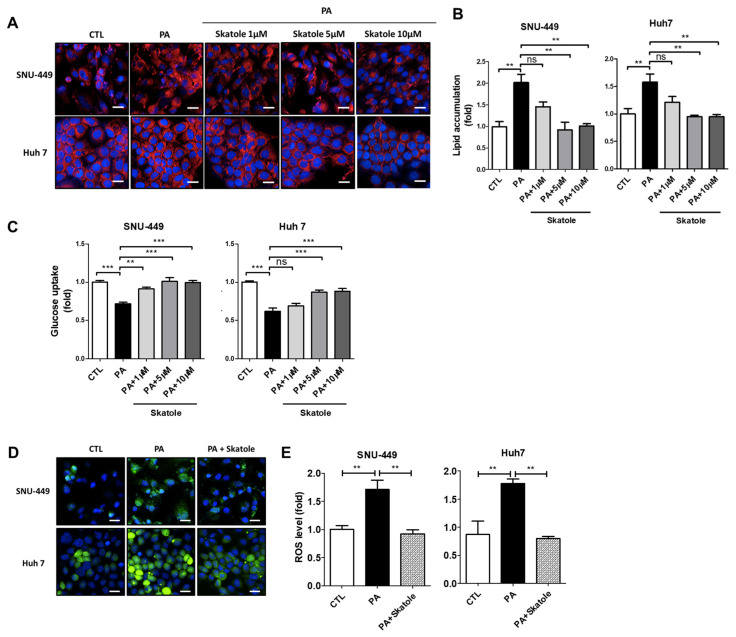
Skatole regulates PA-induced lipid accumulation, glucose uptake, and ROS production in SNU-449 and Huh7 cells. (**A**) Lipid accumulation of SNU-449 and Huh7 cells. Both cells were treated with 0.25 mM PA and 1–10 μM skatole for 24 h. Scale bar, 25 μM. (**B**) Nile Red fluorescence intensity was measured using Columbus software. (**C**) Glucose uptake level of PA and 1–10 μM skatole-treated SNU-449 and Huh7 cells. The relative glucose uptake levels were evaluated by the measurement of 2-NBDG fluorescence intensity. (**D**) Production of ROS in SNU-449 and Huh7 cells treated with PA and 5 μM skatole. (**E**) Relative ROS production levels were measured using Columbus software. Scale bar, 25 μm. Three independent samples were obtained for all experiments, and the values are shown as mean ± SD, analyzed by one-way ANOVA. (*n* = 3–5, *** *p* < 0.001, ** *p* < 0.01, CTL: control).

As discussed earlier, lipotoxicity caused by FFAs is closely related to impaired lipid accumulation, ROS production, and glucose uptake in hepatocytes. Thus, we evaluated whether skatole could restore these lipotoxicity-related features in SNU-449 and Huh7 cells. Lipid accumulation in SNU-449 and Huh7 cells was downregulated by 5 μM skatole (Figure 5A,B). Furthermore, we confirmed that skatole restored glucose uptake in a concentration-dependent manner, which was previously reduced by PA to normal levels in both SNU-449 and Huh7 cells (Figure 5C), and ROS production was decreased by treatment with 5 μM skatole (Figure 5D,E). These results confirmed that 5 μM skatole treatment has an anti-lipotoxic effect on hepatocytes treated with excessive PA.

## 4. Discussion

Here, we describe the beneficial role of skatole in reducing hepatic damage induced by lipotoxicity in three different hepatocytes, i.e., HepG2, SNU-449, and Huh7 cells. This protective effect involves reduced ER stress, oxidative stress, lipogenesis, caspase activity, and apoptosis. In addition, skatole improved lipotoxicity-induced IR and glucose uptake in hepatocytes. 

In general, it has been found that skatole can act as a ligand for aryl hydrocarbon receptor (AhR) and regulate the expression of specific genes of the cyp family, the target gene of AhR, in several cell lines and primary human cells [51,52,53]. Several reports have indicated that high skatole concentrations are toxic via cyp enzyme activities [54,55,56] and cyp-mediated oxidation induced by skatole [57]. However, in the human colon cancer cell line Caco-2, cell viability decreased with treatment of 250 μM skatole for 72 h, which is a higher dose than was used in our study. In addition, Caco-2 cells exposed to 1000 μM of skatole for 24 h showed an increase in cyp1a1 protein level but no statistically significant change in apoptosis levels [58]. According to another previous study, skatole induced the AhR target gene, cyp1a1, in human primary hepatocytes when treated at 200 μM. However, a low-concentration skatole treatment was reported to have no significant association with AhR signaling. In addition, skatole showed a low affinity for the AhR ligand in Hepa1c1c7, a hepatoma cell line [59]. Considering these previous studies, a low-concentration skatole treatment has an insignificant effect on hepatocyte AhR, cyp enzymes, and cell viability. In line with previous studies, in our study, a low-concentration skatole treatment did not affect hepatocyte cell viability but showed a protective effect. 

In this paper, we describe the protective role of skatole in hepatic lipotoxicity, which is caused by exposure to excess lipids and carbohydrates that damage the liver. Similar to our findings, indole-propionic acid (IPA), a 3-substituted indole-like skatole [60] and one of the microbial-derived metabolites of tryptophan, reduced lipotoxicity during the development of NAFLD and improved insulin sensitivity in diabetic animal models [61,62]. In addition, IPA has been reported to prevent lipotoxicity, hepatic lipid synthesis, and inflammatory factors caused by NAFLD [63,64]. In a previous study, J774A.1 cells treated with 250 or 500 μM of IPA showed reduced inflammation in vitro, and hepatic inflammation and injuries were reduced in rats treated with 20 mg/kg/day of IPA [65]. In this study, we determined the lowest concentration of skatole (5 μM) that shows a protective effect for several types of FFA-induced damage. According to previous studies and our findings, these indole metabolites may modulate hepatic lipotoxicity via various mechanisms in the body and have potential for therapeutic approaches.

Indole metabolites, which are produced by the human gut microbiome, have been reported to regulate the immune response [66], and recent studies have shown that they are associated with NAFLD via the gut–liver axis [67]. Furthermore, a previous study showed that the concentration of indole metabolites in the blood of obese patients with NAFLD was reduced compared to that of healthy subjects [68]. Hence, the concentration of indole metabolites can be regarded as a factor related to NAFLD. Despite this association, the relevance of skatole in hepatic metabolic processes has been limited to studies in pigs [69], and the role of skatole in NAFLD and its metabolic processes in the liver is not fully understood. Lipotoxicity is a promising therapeutic target for various metabolic diseases, such as type 2 diabetes [70], ischemic heart disease, and insulin resistance of skeletal muscle [71]. In addition, recent studies have reported that natural compounds can ameliorate hepatic lipotoxicity [72], and hepatic lipotoxicity is a promising target for the development of therapeutic agents for NAFLD [73]. Therefore, it is important to elucidate the cellular damage mechanisms induced by lipotoxicity and the molecular mechanisms acting on NAFLD for therapeutic approaches against lipotoxicity.

## 5. Conclusions

**Figure 6:**
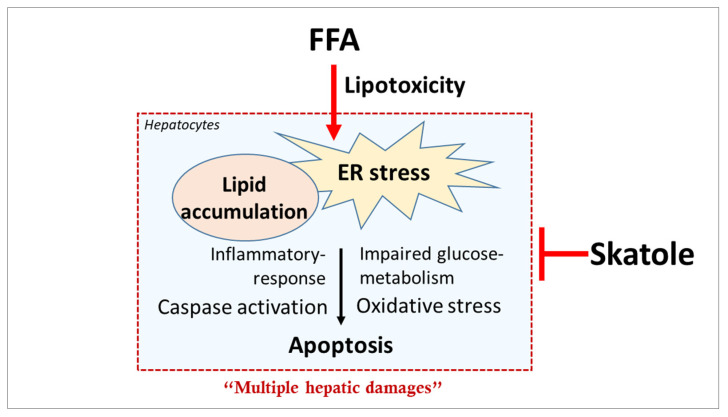
The schematic image represents the protective effect of skatole on hepatocytes.

In conclusion, we elucidated the different roles of the natural product skatole on hepatocytes and confirmed its close involvement in hepatic lipid metabolism (Figure 6). Our study described how skatole, a novel therapeutic target, ameliorates multiple damage mechanisms induced by hepatic lipotoxicity, and our results indicate that skatole can improve lipotoxicity-induced damage in an in vitro NAFLD model. Furthermore, treatment with appropriate concentrations of skatole can have beneficial, protective effects on hepatocytes. However, further animal studies are needed to evaluate the effect of skatole as a therapeutic agent for NAFLD.

## Data Availability

The data that were generated and analyzed for this study are available from the corresponding author upon reasonable request.

## Supplementary Materials

The following supporting information can be downloaded at: https://www.mdpi.com/article/10.3390/nu15061490/s1, Table S1: The list of antibodies for Western blotting.

## Abbreviations

NAFLD, Nonalcoholic fatty liver disease; NASH, Nonalcoholic steatohepatitis; FFAs, Free fatty acids; ER, Endoplasmic reticulum; IR, Insulin resistance; FAS, Fatty Acid Synthase; AKT, Protein kinase B; G6Pase, D-glucose-6-phosphate phosphohydrolase; PCK1, Phosphoenolpyruvate carboxykinase 1; PERK, PKR-like endoplasmic reticulum kinase; IRE1α, Endoplasmic reticulum to nucleus signaling 1; ATF-6, Activating transcription factor 6; BiP, Binding immunoglobulin protein; JNK, c-Jun N-terminal kinases; CHOP, C/EBP homologous protein; eIF2α, eukaryotic initiation factor 2α; MDA, Malondialdehyde; ROS, Reactive oxygen species; TNF- α, Tumor necrosis factor-alpha; Bcl-2, B-cell lymphoma 2; Bax, Bcl-2-associated X protein; PARP, Poly (ADP-ribose) polymerase; IL-6, Interleukin 6; p38, p38 mitogen-activated protein kinases; AhR, aryl hydrocarbon receptor; IPA, indole-propionic acid.
