# Supplementary material for: Natural Product Skatole Ameliorates Lipotoxicity-Induced Multiple Hepatic Damage under Hyperlipidemic Conditions in Hepatocytes
Source: Nutrients. 2023 Mar 20;15(6):1490. doi: 10.3390/nu15061490 (PMC10052055; doi:10.3390/nu15061490)
Supplement: Supplementary file 1 [file nutrients-15-01490-s001.zip › nutrients-2267895-supplementary.pdf]

Table S1. The list of antibodies for western blotting.

Antibody	Company	Cat No
β-Actin (13E5)	Cell Signaling Technology	4970
Fatty Acid Synthase (C20G5)	Cell Signaling Technology	3180
Lipin-1 (H-120)	Santa Cruz Biotechnology	sc-98450
Phospho-Akt (Ser473)	Cell Signaling Technology	4060
AKT	Cell Signaling Technology	9272
G6Pase-a (H-60)	Santa Cruz Biotechnology	sc-25840
PCK1 (D12F5)	Cell Signaling Technology	12940
BiP (C50B12)	Cell Signaling Technology	3177
CHOP (L63F7)	Cell Signaling Technology	2895
ATF-6 (D4Z8V)	Cell Signaling Technology	65880
IRE1α (14C10)	Cell Signaling Technology	3294
PERK (C33E10)	Cell Signaling Technology	3192
SAPK/JNK	Cell Signaling Technology	9252
Phospho-SAPK/JNK (Thr183/Tyr185)	Cell Signaling Technology	9251
eIF2α (D7D3)	Cell Signaling Technology	5324
Phospho-eIF2α (Ser51) (119A11)	Cell Signaling Technology	3597
PARP (46D11)	Cell Signaling Technology	9532
Caspase-3	Cell Signaling Technology	9662
Cleaved caspase-3 (Asp175)	Cell Signaling Technology	9661
Bcl-2	Cell Signaling Technology	2876
Bax	Cell Signaling Technology	2772
TNF-α	Abcam	ab6671
IL-6	Santa Cruz Biotechnology	sc-57315
p38 MAPK (D13E1)	Cell Signaling Technology	8690
Phospho-p38 MAPK (Thr180/Tyr182)	Cell Signaling Technology	9211
